# Supplementary figures and images for: Technical Report on the New Ultrasound Lateral Mid-Shaft Approach to the Sciatic Nerve: A Never-Ending Story
Source: Medicina (Kaunas). 2025 Jan 10;61(1):100. doi: 10.3390/medicina61010100 (PMC11767092; doi:10.3390/medicina61010100)

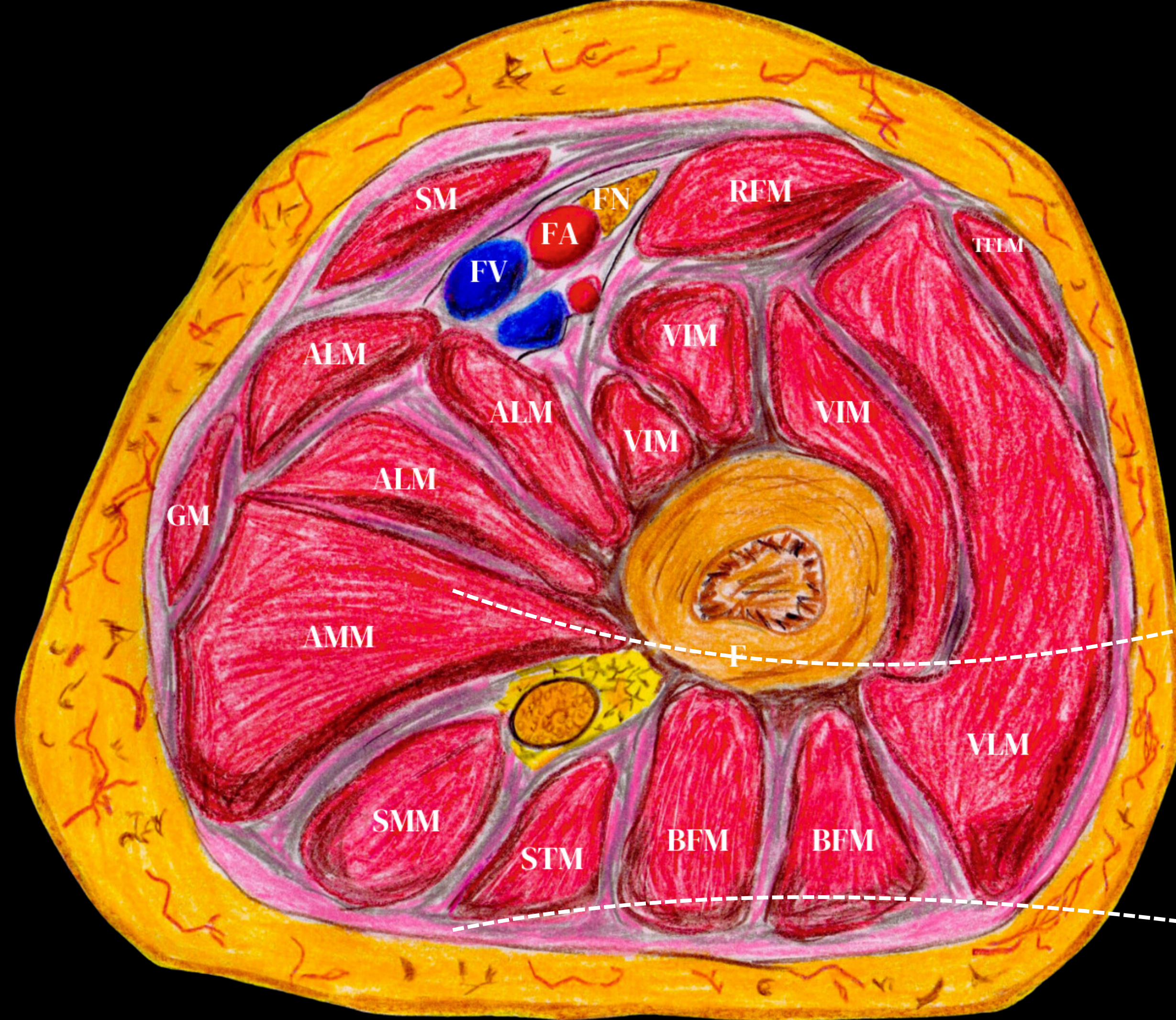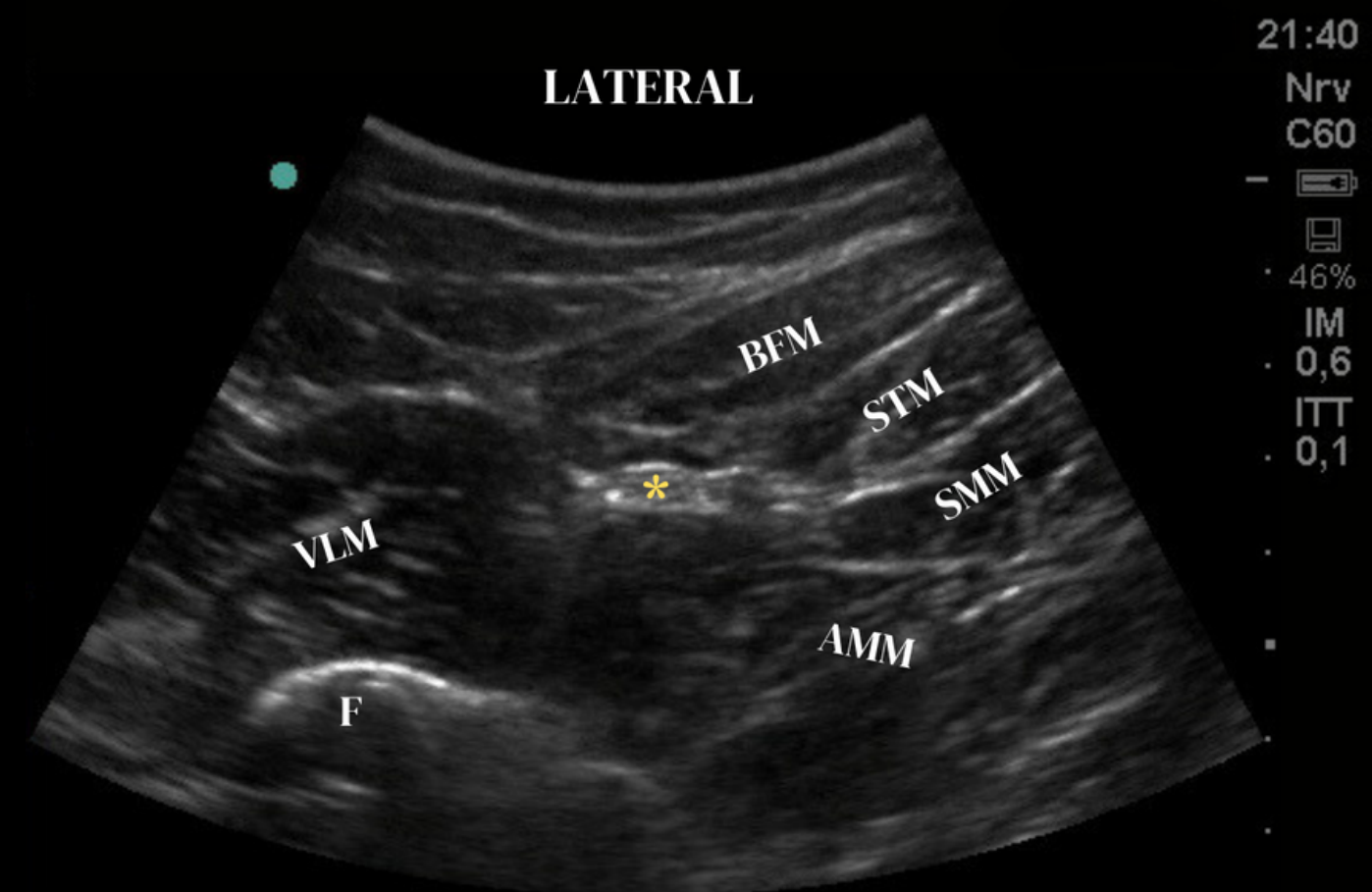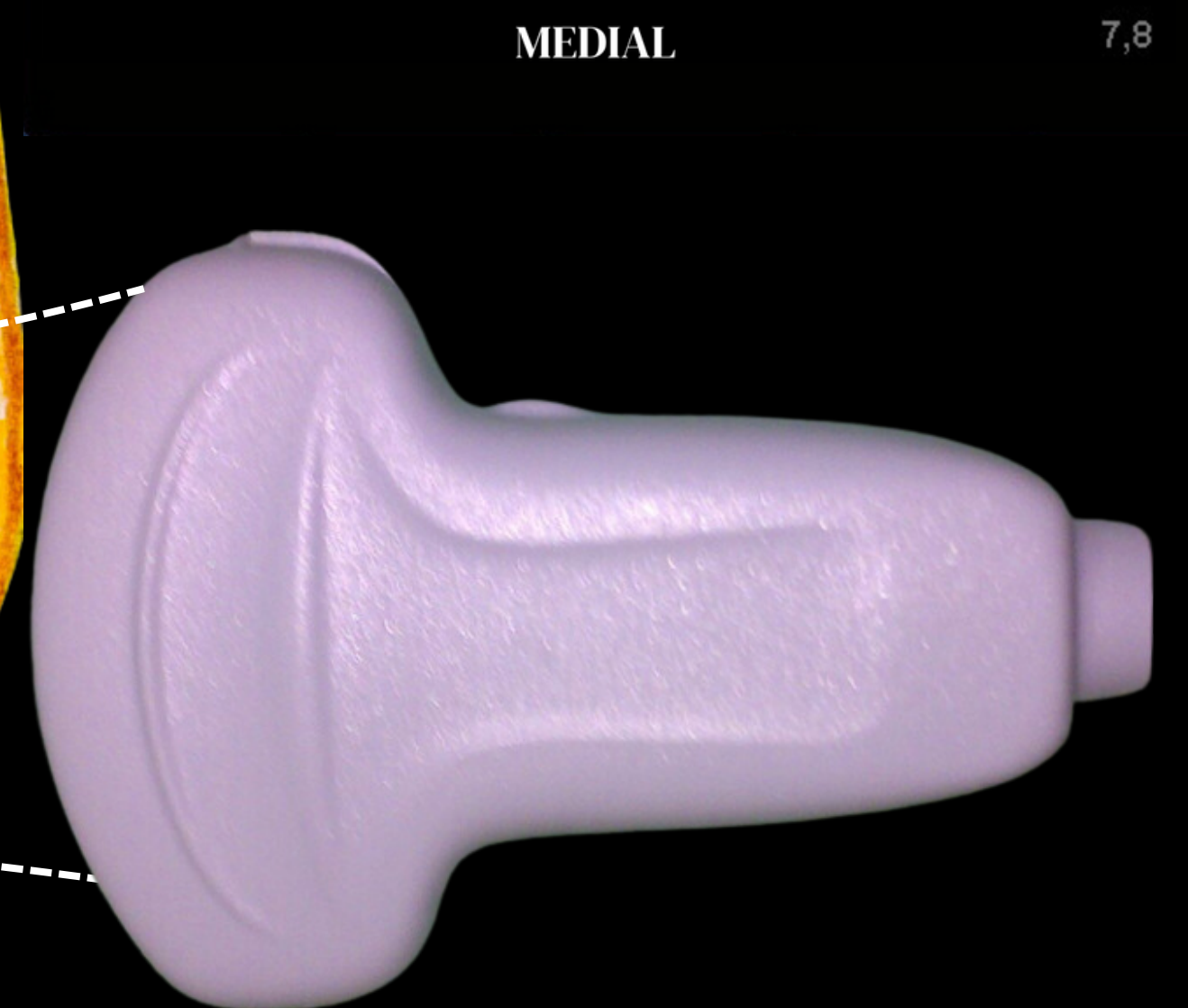

Supplement: Supplementary file 1 [file medicina-61-00100-s001.zip › medicina-3393209-supplementary/Figure S1.pdf]

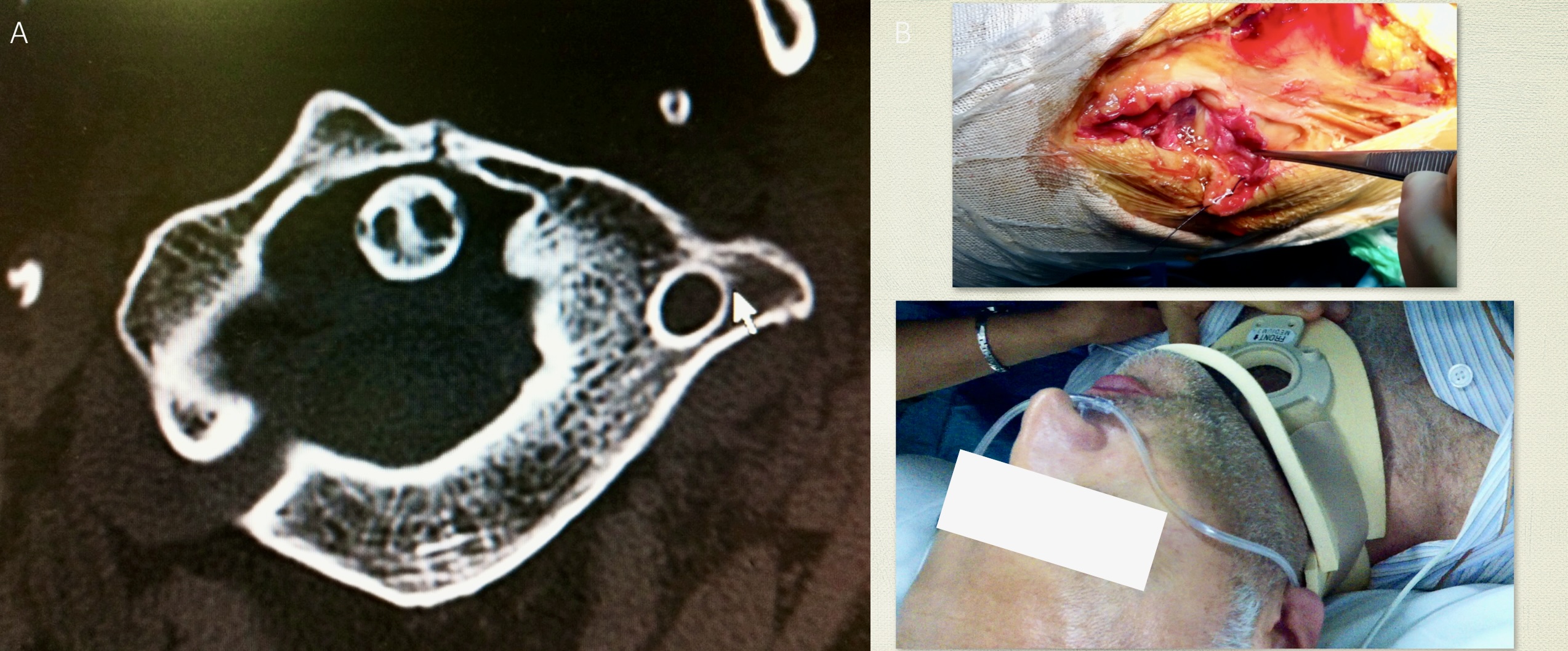

Supplement: Supplementary file 1 [file medicina-61-00100-s001.zip › medicina-3393209-supplementary/Figure S2.jpeg]
